# Formation and Morphology of Zn_2_Ti_3_O_8_ Powders Using Hydrothermal Process without Dispersant Agent or Mineralizer

**DOI:** 10.3390/ijms12020935

**Published:** 2011-01-28

**Authors:** Cheng-Li Wang, Weng-Sing Hwang, Kuo-Ming Chang, Horng-Huey Ko, Chi-Shiung Hsi, Hong-Hsin Huang, Moo-Chin Wang

**Affiliations:** 1 Department of Materials Science and Engineering, National Cheng Kung University, 1 Ta-Hsueh Road, Tainan 70101, Taiwan; E-Mails: N58991223@ncku.edu.tw (C.-L.W.); wshwang@mail.ncku.edu.tw (W.-S.H.); 2 Department of Mechanical Engineering, National Kaohsiung University of Applied Sciences, 415 Chien-Kung Road, Kaohsiung 80782, Taiwan; E-Mail: koming@cc.kuas.edu.tw; 3 Department of Fragrance and Cosmetics Science, Kaohsiung Medical University, 100 Shih-Chuan 1^st^ Road, Kaohsiung 80708, Taiwan; E-Mail: hhko@kmu.edu.tw; 4 Department of Materials Science and Engineering, National United University, 1 Lein-Da, Kung-Ching Li, Miao-Li 36003, Taiwan; E-Mail: chsi@nuu.edu.tw; 5 Department of Electrical Engineering, Cheng Shiu University, 840, Cheng Ching Road, Niaosong, Kaohsiung 83347, Taiwan; E-Mail: funs@csu.edu.tw

**Keywords:** Zn_2_Ti_3_O_8_ powders, hydrothermal, UVA-attenuating

## Abstract

Synthesis of Zn_2_Ti_3_O_8_ powders for attenuating UVA using TiCl_4_, Zn(NO_3_)_2_·6H_2_O and NH_4_OH as precursor materials by hydrothermal process has been investigated. The X-ray diffractometry (XRD) results show the phases of ZnO, anatase TiO_2_ and Zn_2_Ti_3_O_8_ coexisted when the zinc titanate powders were calcined at 600 °C for 1 h. When calcined at 900 °C for 1 h, the XRD results reveal the existence of ZnO, Zn_2_TiO_4_, rutile TiO_2_ and ZnTiO_3_. Scanning electron microscope (SEM) observations show extensive large agglomeration in the samples. Transmission electron microscope (TEM) and electron diffraction (ED) examination results indicate that ZnTiO_3_ crystallites formed with a size of about 5 nm on the matrix of plate-like ZnO when calcined at 700 °C for 1 h. The calcination samples have acceptable absorbance at a wavelength of 400 nm, indicating that the zinc titanate precursor powders calcined at 700 °C for 1 h can be used as an UVA-attenuating agent.

## 1. Introduction

Ultraviolet (UV) radiation that reaches the earth and damages skin can be divided into three key wavelengths: (i) UVC (32–280 nm), (ii) UVB (280–320 nm) and (iii) UVA (320–400 nm). UVA radiation is a major culprit in photoaging and skin cancers. Moreover, UVB, which primarily reaches the top-most layer of skin, is thought to be responsible for acute photodamage, including sunburn and some non-melanoma skin cancers [1]. Therefore, protection against harmful UVA and UVB radiation is very important. Sheath [2] pointed out that sunscreens used for the protection of human skin against the harmful effects of solar radiation must contain UV-absorbing substances.

Fine particles of various metal oxides, such ZnO and TiO_2_, are extensively used as agents to attenuate (scatter and/or absorb) UV radiation, and have many attractive characteristics, such as a long history of topical use, broad spectrum absorption, high photostability and low irritancy [3]. However, an extensive literature search found that the use of ZnO-TiO_2_ as a sunscreen for cosmetic applications has not been fully investigated.

Dulin and Rase [4] first established the basic phase diagram of the ZnO-TiO_2_ system, and reported the temperature and composition ranges of stability for zinc metatitanate (ZnTiO_3_) and zinc orthotitanate (Zn_2_TiO_4_). Only Zn_2_Ti_3_O_8_, ZnTiO_3_ and Zn_2_TiO_4_ have been confirmed to exist in ZnO-TiO_2_ systems by previous researchers [4–7]. The compound of Zn_2_Ti_3_O_8_ has a cubic structure with a lattice constant of *a*_o_ = 0.8390(5) nm, and has been observed to be a low-temperature form of ZnTiO_3_ that exists at temperatures below 820 °C [7]. ZnTiO_3_ has a rhombohedral structure with lattice constants of *a*_o_ = 0.5078(2) and *c*_o_ = 1.3920(1) nm [5]. When heated between 965 and 1010 °C, ZnTiO_3_ decomposes and forms Zn_2_TiO_4_ and rutile TiO_2_ [6]. The compound of Zn_2_TiO_4_ has a face-centered cubic crystal structure with a lattice constant of *a*_o_ = 0.8460(2) nm [8].

Zinc titanates, such as ZnTiO_3_ and Zn_2_TiO_4_, are attractive as sorbents for removing sulfur from hot coal gasification products [9,10], pigments [11], and gas sensors for ethanol, NO and CO [12]. Due to the recent progress of microwave applications in the area of mobile telephones and satellite communications, these substances can also be used as dielectric resonators and fitters [13,14]. Furthermore, Chang *et al*. [15,16] also found that doped and undoped ZnTiO_3_ have a V-type resistivity-temperature characteristic and possess typical positive thermal coefficient resistivity (PTCR) properties when above the transition point. However, the use of Zn_2_Ti_3_O_8_ or ZnTiO_3_ as UV-attenuating agents has not been reported.

The chemistry and microstructure are important factors for applications of zinc titanate powders. Hence, various methods have been adopted for the preparation of ZnTiO_3_ powders, including conventional solid state reaction [5] and sol-gel processes [16,17]. In addition, Zn_2_TiO_4_ powders have been obtained by solid-state reaction [8], and the ball mill method [18]. However, the solid-state reaction processes have some drawbacks, such as high reaction temperature, large particle size and limited degree of chemical homogeneity. On the other hand, Reddy *et al*. [19] pointed out that a single phase of Zn_2_Ti_3_O_8_ is produced when zinc titanyl oxalate hydrate decomposes at 650 °C for several hours. However, until now, no information is available on the synthesis and characterization of Zn_2_Ti_3_O_8_ powders by a hydrothermal process without the addition of a dispersant agent.

In the present study, high purity TiCl_4_ and Zn(NO_3_)_2_·6H_2_O have been used for the synthesis of Zn_2_Ti_3_O_8_ crystallite powders by a hydrothermal process without the addition of either a dispersant agent or mineralizer. The main purpose of the present investigation was to examine the formation and morphology of Zn_2_Ti_3_O_8_ nanocrystallite powders. In addition, this study (i) investigated the thermal behavior of zinc titanate precursor powders, (ii) evaluated the phase transition of zinc titanate precursor powders, and (iii) observed the morphology of zinc titanate precursor powders after calcination at various temperatures for 1 h.

## 2. Experimental Procedure

### 2.1. Sample Preparation

The Zn_2_Ti_3_O_8_ nanocrystallite powders were prepared by a hydrothermal process without the addition of a dispersant agent. The starting materials were prepared in a aqueous solution with reagent-grade titanium tetrachloride solution (TiCl_4_, purity ≥ 98.0%, supplied by Fulka, France), zinc nitrate (Zn(NO_3_)_2_·6H_2_O, purity ≥ 98.0%, supplied by Slfa Aersor, USA) and 25 vol% ammonia solution (NH_4_OH, supplied by Riedel-de Haën, Germany). 0.05 M and 1.0 vol% aqueous solutions were prepared from reagent-grade TiCl_4_, Zn(NO_3_)_2_·6H_2_O and 25 vol% NH_4_OH, respectively. A molar ratio of [Zn^2+^]/[Ti^4+^] was 1.0. An aqueous solution of Zn(NO_3_)_2_ was added to TiCl_4_ solution under an air atmosphere. The pH of the mixture was then raised to 7.0 by using NH_4_OH aqueous solution and stirring the resulting solution for 2 h at room temperature. Subsequently, the solution was kept in an autoclave at 150 °C for 1 h. After cooling, the precipitates obtained were filtered, and washed thoroughly three times with a large amount of deionized water and ethanol (purity ≥ 99.85%, supplied by J. J. Baker, USA) to remove Cl^−^. The final precipitates were dried at −55 °C in a vacuum and the white zinc titanate precursor powders were thus obtained.

### 2.2. Sample Characterization

Differential thermal analysis (DTA, Perkin-Elmer 7 Series Thermal Analysis System, Boston, MA, USA) was conducted on 50 mg zinc titanate precursor powders at a heating rate of 10 °C/min in air with a reference material of Al_2_O_3_. The calcination temperature was determined from the DTA result.

The crystalline phase was identified using an X-ray diffractometer (XRD, Rigaku D-Max**/**IIIV, Tokyo, Japan) with Cu K*_α_* radiation and Ni filter, operated at 30 kV, 20 mA and a scanning rate of 0.25°/min.

The morphology of the zinc titanate precursor powders calcined at various temperatures for 1 h were observed with a scanning electron microscope (SEM, Hitachi, S-3000N, Japan) and transmission electron microscope (TEM, Hitachi model HF-2000, Tokyo, Japan). The crystal structure of the post-calcined powders was determined by selected area electron diffraction (SAED) analysis. The TEM samples were prepared by dispersing the post-calcined powders in an ultrasonic bath and then collected on a copper grid.

## 3. Results and Discussion

### 3.1. Thermal Behavior of the Zinc Titanate Precursor Powders

**Figure 1.**
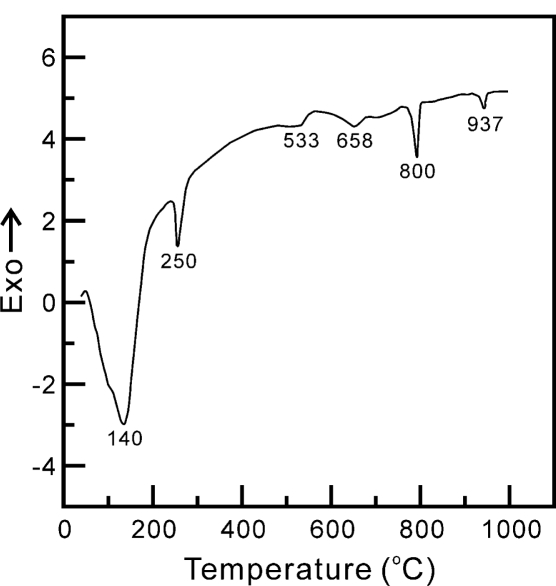
Differential thermal analysis (DTA) curve of zinc titanate precursor powders with a heating rate of 10 °C·min^−1^.

The DTA curve of the zinc titanate precursor powders, produced without the addition of either a dispersant agent or mineralizer, and which was heated from 25 to 1000 °C in static air at a heating rate of 10 °C/min, is shown in Figure 1. There are four endothermic peaks at 140, 250, 800 and 940 °C in the DTA curve. The endothermic peak at 140 °C is due to the dehydration of the zinc titanate precursor powders. The second endothermic peak, at 250 °C, is attributed to the decomposition of NH_2_- into N_2_ and H_2_ [20]. The third endothermic peak, at 800 °C, is caused by the decomposition of Zn_2_Ti_3_O_8_ into ZnTiO_3_ and rutile TiO_2_. The fourth endothermic peak, at 940 °C, is due to the ZnTiO_3_ decomposing, which leads to the formation of Zn_2_TiO_4_ and rutile TiO_2_. Moreover, Figure 1 also shows two relatively small broad exothermic peaks at around 558 and 689 °C. The first exothermic peak, at 558 °C, is due to the anatase TiO_2_ accompanied by Zn_2_Ti_3_O_8_ formation. The second exothermic peak at 689 °C is caused by the ZnTiO_3_ accompanied with rutile TiO_2_ formation.

### 3.2. Phase Transition of Zinc Titanate Precursor Powders Calcined at Various Temperatures for 1 h

**Figure 2.**
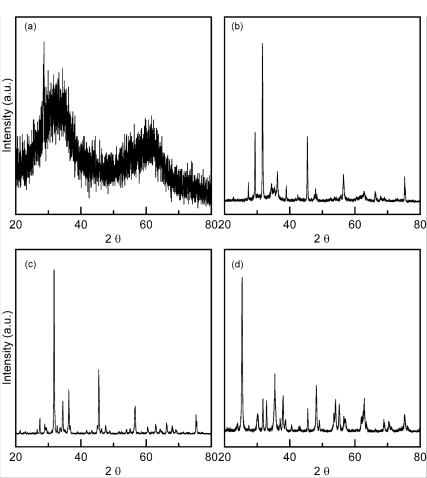
X-ray diffraction (XRD) patterns of ZnTiO_3_ precipitates calcined at various temperatures for 1 h: (**a**) before calcination, (**b**) calcined at 600 °C, (**c**) 700 °C, and (**d**) 900 °C.

Figure 2 shows the XRD patterns of the zinc titanate precursor powders prepared without a dispersant agent or mineralizer and calcined at various temperatures for 1 h. The XRD pattern of the freeze dried precursor powders before calcination is shown in Figure 2(a), which reveals that the precursor powders still maintained the amorphous state. Figure 2(b) shows the XRD pattern of the zinc titanate precursor powders calcined at 600 °C for 1 h, and indicates that the anatase TiO_2_ appeared due to the reflections located (101), (110), (103), (200), (105), (211) and (220) (JCPDS Cards No.89-4203). Figure 2(b) also shows the presence of ZnO, due to the reflection peaks located at (100), (110) and (103) (JCPDS Card No.89-1397). Furthermore, the reflection peaks of Zn_2_Ti_3_O_8_ also appeared at (210), (220), (400), (440), and (622) (JCPDS Card No.87-1991). The XRD pattern of zinc titanate precursor powders calcined at 700 °C for 1 h are illustrated in Figure 2(c), which reveals that the crystallized phases were composed of the major phases of ZnO and Zn_2_Ti_3_O_8_, with rutile TiO_2_ as the secondary phase and the minor phases of ZnTiO_3_ and anatase TiO_2_. Figure 2(d) shows the XRD pattern of zinc titanate precursor calcined at 900 °C for 1 h. It reveals that the crystallized phase was composed of ZnO, Zn_2_TiO_4_, rutile TiO_2_ and ZnTiO_3_, but the anatase TiO_2_ disappeared.

Moreover, from Figure 2(c) and (d), it is seen that there is a significantly higher intensity value for the ZnO (100) reflection (I_100_). Golón *et al*. [21] have pointed out that for hydrothermal treatment systems, samples reveal an apparent preferential orientation growth in the (100) direction, leading to a significant I_100_ value. In fact, zinc and oxygen atoms are arranged alternatively along the c-axis, and thus as is well established, this inherent asymmetry along the c-axis results in the anisotropic growth of ZnO crystallites.

On the other hand, Bartram and Slepetys [5] pointed out that with a sample prepared at the mole ratio of ZnO:TiO_2_ = 2:1 and calcined at 700 and 800 °C for various times, the phase of defect-spinel type Zn_2_Ti_3_O_8_ with a trace amount of uncombined TiO_2_ is produced. This is caused by the four Ti ions that are missing from the 16-point positions of the spinel-type structure arrangement, resulting in a defective spinel-type structure. Mrázek *et al*. [22] reported that the TiO_2_ and Zn_2_TiO_4_ are decomposed from prepared Zn_x_Ti_y_O_z_ powders for various ratios of ZnO/TiO_2_. TiO_2_ exists at temperatures of 400–600 °C prepared by sol-gel method [23].

In Figure 2(b) and (c), it can be seen that although the intensity of Zn_2_Ti_3_O_8_ increases with the calcination temperature, a small fraction of Zn_2_Ti_3_O_8_ decomposes and leads to the formation of the ZnTiO_3_ and rutile TiO_2_. This reaction can be expressed as follows:

(1) $${\text{Zn}}_{2}{\text{T}}_{3}{\text{O}}_{8}\to 2\text{Zn}{\text{TiO}}_{3}+{\text{TiO}}_{2(\text{r})}.$$

Yang and Swisher [24] also pointed out that Zn_2_Ti_3_O_8_ is a thermodynamically stable compound up to temperatures between 700 and 800 °C. Just above this temperature, ZnTiO_3_ is more stable than the compound of Zn_2_Ti_3_O_8_. Furthermore, Yamaguchi *et al*. [6] also proposed using an amorphous material prepared by the simultaneous hydrolysis of zinc acetylautonate and titanium isopropoxide for synthesis of the ZnTiO_3_ powders. The XRD result shows the reflection peaks of the compound corresponding to Zn_2_Ti_3_O_8_ appeared at 600 °C [5] and the intensity of the reflection peaks increased rapidly up to 760 °C. No other compounds and free species, except for the hexagonal form of ZnTiO_3_, are observed up to the decomposition temperature at 965 °C. These results suggest that the compound so far denoted as Zn_2_Ti_3_O_8_ is a low temperature form of ZnTiO_3_.

On the other hand, the phase of Zn_2_TiO_4_ formed by the thermal decomposition of Zn_2_Ti_3_O_8_ in the range of 650–900 °C has been reported by previous studies [5,25]. Figure 2(c) shows that for the zinc titanate precursor powders calcined at 700 °C for 1 h, only a small fraction of Zn_2_Ti_3_O_8_ decomposed and formed the ZnTiO_3_ and rutile TiO_2_. This result was attributed to the fact that the phase of Zn_2_Ti_3_O_8_ at 700 °C still has thermal stability. When calcined at 900 °C for 1 h, the Zn_2_Ti_3_O_8_ phase disappeared and the reflection peaks of ZnTiO_3_ also nearly vanished, but the intensity of Zn_2_TiO_4_ and rutile TiO_2_ increased. This is because the Zn_2_Ti_3_O_8_ and ZnTiO_3_ phases decomposed, leading to the formation of Zn_2_TiO_4_ and rutile TiO_2_. These reactions can be expressed as follows:

(2) $${\text{Zn}}_{2}{\text{Ti}}_{3}{\text{O}}_{8}\to {\text{Zn}}_{2}{\text{TiO}}_{4}+2{\text{TiO}}_{2(\text{r})}$$

(3) $$2\text{Zn}{\text{TiO}}_{3}\to {\text{Zn}}_{2}{\text{TiO}}_{4}+{\text{TiO}}_{2(\text{r})}$$

### 3.3. Microstructure of the Zinc Titanate Precursor Powders Calcined at Various Temperatures for 1 h

**Figure 3.**
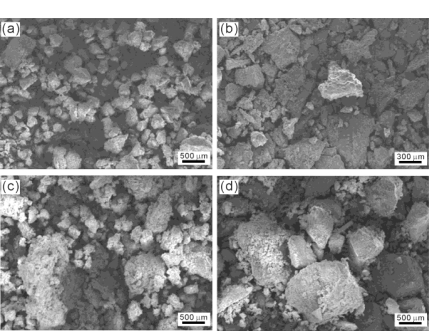
Scanning electron microscope (SEM) morphology of zinc titanate precursor powders calcined at various temperatures for 1 h: (**a**) before calcination, (**b**) calcined at 600 °C, (**c**) 700 °C, and (**d**) 900 °C.

The SEM microstructure of the zinc titanate precursor powders calcined at various temperatures for 1 h are shown in Figure 3. Figure 3(a) shows the morphology of the freeze-dried zinc titanate precursor powders without a dispersant agent or mineralizer agglomerates to the size of about 140 ± 70 μm. The SEM micrographs in Figure 3(b) and (d) shows the zinc titanate precursor powders calcined at 600, 700 and 900 °C for 1 h, respectively. It can be seen that the agglomerated size of the particles increases as the calcination temperature rises from 600 to 900 °C. When calcined at 900 °C for 1 h, the size increases from 140 ± 70 μm to 270 ± 170 μm. Since the zinc titanate precursor powders were prepared through the wet-chemical routes, during this process, *i.e.*, drying and/or subsequent steps, agglomeration can occur. During calcination, the most common type of agglomeration in the conventional powders was due to solid bonds that formed between the particles.

**Figure 4.**
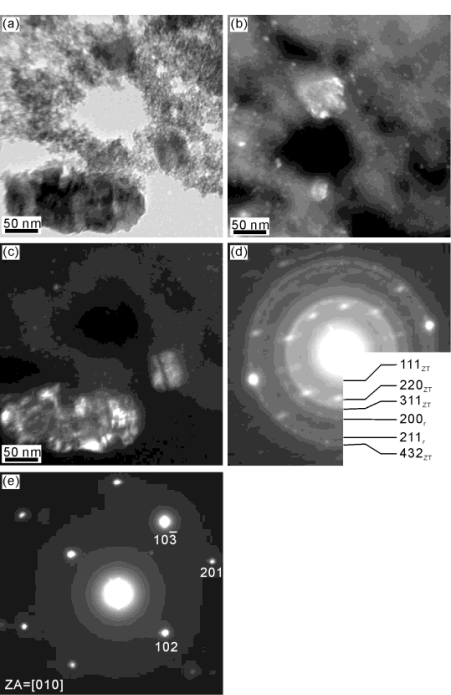
Transmission electron microscope (TEM) morphology and electron diffraction (ED) patterns of zinc titanate precursor powders calcined at 700 °C for 1 h: (**a**) bright field (BF) image, (**b**) dark field (DF) image by using a circle spot of (**d**), (**c**) DF image by using a circle spot of (**e**), (**d**) ED pattern corresponding to the phases of Zn_2_Ti_3_O_8_ (denoted by ZT) and rutile TiO_2_ (denoted by r), and (**e**) ED pattern corresponding to ZnO.

The bright field (BF) and dark field (DF) TEM micrographs and the corresponding electron diffraction (ED) patterns of the freeze dried zinc titanate precursor powders calcined at 700 °C for 1 h are shown in Figure 4. Figure 4(a) shows the BF image, in which fine particles with size of about 5 nm and a larger particle with a length of 200 nm and width of 100 nm are observed. Aubert *et al*. [26] also reported the particle of TiO_2_ is about 5 nm. Figure 4(a) shows that the larger particles, of ZnO which cause the contact area of ZnO with anatase TiO_2_ to decrease, led to a decrease in the reaction of ZnO with anatase TiO_2_, meaning that insufficient Zn_2_Ti_3_O_8_ was produced. Figure 4(b),(c) shows the DF images of the fine and larger particles in Figure 4(a). In addition, Figure 4(d),(e) shows the ED patterns of the particles in Figure 3(b),(c), respectively. The ED pattern of Figure 4(d) corresponds to the phases of ZnTiO_3_ and rutile TiO_2_. On the other hand, the ED pattern of Figure 4(e) corresponds to ZnO. Figure 4(d) also shows evidence of ZnTiO_3_ and rutile TiO_2_ when calcined at 700 °C for 1 h. Moreover, the microstructure of the ZnTiO_3_ crystallite shows a nearly spherical morphology growing on the matrix of a plate-like phase of ZnO.

**Figure 5.**
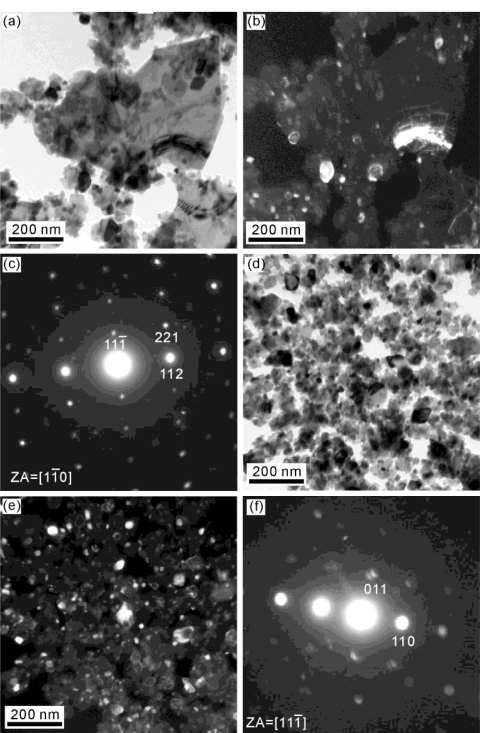
TEM morphology and ED patterns of zinc titanate precursor powders calcined at 900 °C for 1 h: (**a**) BF image, (**b**) DF image by using a circle spot of (**c**), (**c**) ED pattern corresponding to ZnTiO_2_, (**d**) BF image, (**e**) DF image by using a circle spot of (**f**), and (**f**) ED pattern corresponding to Zn_2_TiO_3_.

Figure 5(a),(b) shows the BF and DF images of the freeze dried zinc titanate precursor powders calcined at 900°C for 1 h, revealing that two kinds morphology coexist in the sample. One is the fine particles with a size of about 50 nm, and the other one is belt-shape particles with a length of 200 nm and width of 50 nm. The ED pattern of the Figure 5(c) belt-shaped and fine particles correspond to ZnTiO_3_ with a zone axis (ZA) of [110]. Figure 5(d),(e) shows the BF and DF images of the fine particles with the size of about 38 ± 18 μm. The ED pattern of Figure 5(f) corresponds to ZnTiO_3_ with the ZA of [ıı̄ı].

### 3.4. The Transmittance of Zinc Titanate Precursor Powders Calcined at 900 °C for 1 h

**Figure 6.**
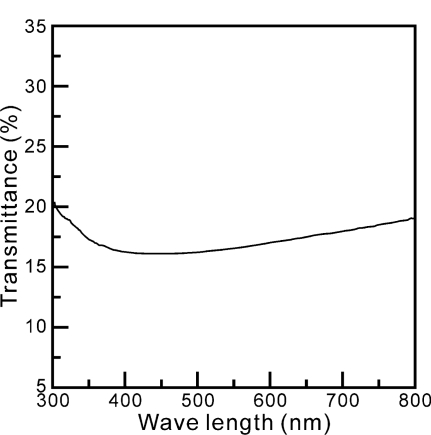
Relation between the absorbed and wavelength between 300 and 800 nm for ZnTiO_3_ precipitates calcined at 900 °C for 1 h.

Figure 6 shows the relation between transmittance and wavelength range between 300 and 800 nm for freeze dried zinc precursor powders calcined at 700 °C for 1 h. It is found that the calcined sample has an acceptable transmittance at the wavelength of 400 nm. This result indicates that zinc titanate precursor powders calcined at 700 °C for 1 h can be used as an UVA-attenuating agent.

## 4. Conclusions

Zn_2_Ti_3_O_8_ powders prepared by a hydrothermal method without a dispersant agent or mineralizer for use in UVA-attenuating applications have been investigated using DTA, XRD, SEM, TEM, ED and UV/VIS. The results are summarized as follows:

1. When the zinc titanate precursor powders were prepared at pH = 7 and calcined at 600 °C for 1 h, the XRD results show that the phases of ZnO, anatase TiO_2_ and Zn_2_Ti_3_O_8_ coexisted in the sample. However, when calcined at 900 °C for 1 h, the XRD result reveals the existence of Zn_2_TiO_4_, rutile TiO_2_, and ZnO.
2. The SEM results reveal significant agglomeration in both the freeze-dried and post-calcined samples.
3. The TEM and ED examination indicates the existence of near spherical Zn_2_Ti_3_O_8_ crystallites with size of about 5 nm on larger ZnO particles with a length of 200 nm and width of 100 nm. The microstructure ZnTiO_3_ shows a somewhat belt-shaped morphology, with a length of 200 nm and width of 50 nm for precipitates calcined at 900 °C for 1 h.
4. The calcined samples have an acceptable transmittance when the wavelength is 400 nm. This result indicates that zinc titanate precursor powders calcined at 700 °C for 1 h can be used as an UVA-attenuating agent.
